# LAPAROSCOPIC PERITONEAL DIALYSIS CATHETER PLACEMENT WITH RECTUS SHEATH TUNNELING: A ONE-PORT SIMPLIFIED TECHNIQUE

**DOI:** 10.1590/0102-672020220002e1690

**Published:** 2022-09-16

**Authors:** Ana Carolina Buffara BLITZKOW, Gilson BIAGINI, Carlos Antonio SABBAG, Victor Assad BUFFARA-JUNIOR

**Affiliations:** 1 Paraná Kidney Institute, Peritoneal Dialysis Service - Curitiba (PR), Brazil;; 2 Santa Cruz Hospital, General Surgery Department - Curitiba (PR), Brazil;; 3 Pilar Hospital, General Surgery Department - Curitiba (PR), Brazil.

**Keywords:** Peritoneal Dialysis, Laparoscopy, Catheters, Outpatients, General Surgery, Diálise Peritoneal, Laparoscopia, Cateteres, Pacientes Ambulatoriais, Cirurgia Geral

## Abstract

The success of peritoneal dialysis depends on the proper placement and functional longevity of the dialysis catheter. Laparoscopic implantation of a catheter through a rectus sheath tunneling can minimize the risks of catheter failure.

**AIMS:**

This study aims to describe one-port simplified technique for laparoscopic placement of a peritoneal dialysis catheter with rectus sheath tunneling.

**METHODS:**

The simplified laparoscopic insertion of a Tenckhoff catheter with rectus sheath tunneling was performed in 16 patients with chronic renal failure.

**RESULTS:**

During the follow-up period, no major complications occurred. Three patients were excluded. One was referred to the renal transplant some weeks after implantation, and one died for other reasons during the follow-up. Another patient needed adhesiolysis due to previous surgery, so an additional port was necessary. The other 13 catheters worked properly, and no postoperative hemorrhage, early leaks, hernia, or catheter migration occurred. One patient had a tunnel infection 11 months after the implant. No peritonitis was observed during the follow-up.

**CONCLUSIONS:**

The technique is simple, reproducible, and safe, with good results in catheter function, few complications, and a high catheter survival rate. It does not require a special device or trocar and avoids excessive port sites.

## RESUMO - RACIONAL

O sucesso da diálise peritoneal depende da implantação adequada e da longevidade funcional do cateter. O implante laparoscópico através da tunelização na bainha do reto abdominal minimiza os riscos de disfunção do cateter.

**OBJETIVOS:**

Descrever técnica simplificada com portal único para o implante laparoscópico de cateter de diálise peritoneal com tunelização na bainha do reto abdominal.

**MÉTODOS:**

Utilizou-se inserção laparoscópica de cateter de Tenckhoff com tunelização da bainha do reto em 16 pacientes com insuficiência renal crônica.

**RESULTADOS:**

Durante o período de acompanhamento não ocorreram complicações relacionadas ao procedimento. Três pacientes foram excluídos: um foi encaminhado para transplante renal algumas semanas após o implante e outro faleceu por outro motivo durante o acompanhamento. Um terceiro necessitou de lise de aderências devido à operação anterior, portanto foi necessário um portal adicional. Os outros 13 pacientes apresentaram bom funcionamento do cateter. Não houve hemorragia pós-operatória, vazamentos, hérnia ou migração do cateter. Um paciente teve infecção no túnel subcutâneo 11 meses após o implante. Não foi observada peritonite durante o período de acompanhamento.

**CONCLUSÕES:**

A técnica é simples, reprodutível, segura, com bons resultados de funcionalidade, poucas complicações e alta taxa de sobrevida do cateter. Ela não requer trocarte especial e evita o uso excessivo de portais.

## INTRODUCTION

Continuous ambulatory peritoneal dialysis (PD) is an effective form of treatment for patients with end-stage renal disease^22^. Successful PD treatment depends on the proper insertion and functional longevity of the dialysis catheter. Knowledge of best practices in catheter insertion can minimize the risk of complications that can lead to PD failure^20^.

The introduction of laparoscopy brought great advances in the implantation of PD catheters. The percentage of PD implanted laparoscopically in the USA has almost doubled from 26% in 2007 to nearly 50% in 2012^7^ Some laparoscopic insertion techniques described aimed to minimize the risk of catheter failure. The placement of a PD catheter through a rectus sheath tunnel (RST) can reduce complications and improve catheter survival^9^ ^,^ ^10^ ^,^ ^26^.

There are no recent publications in our country about the laparoscopic aspects of peritoneal catheter implants. The aim of this study was to present a reproducible technique for laparoscopic placement of a PD catheter with rectus sheath tunneling using only a 10 mm port.

## METHODS

The study was approved by the local Research Ethics Committee, and all the patients signed informed consent before the surgery. The contraindications for the technique were those related to general anesthesia and pneumoperitoneum. We enrolled 16 patients with end-stage renal disease from March 2018 to March 2020 at two hospitals in the city of Curitiba, PR, Brazil.

### Surgical Technique

Antibiotic prophylaxis with cefazolin 1 g intravenous is administered. Patients are placed in the dorsal decubitus position under general anesthesia. Laparoscopic access to the abdominal cavity is performed by Veress needle insertion technique, supraumbilical, 10 mm incision, followed by the introduction of a trocar and a 30° scope. Carbon dioxide gas insufflation is about 10-12 mmHg. An abdominal cavity inspection is made.

**Figure 1 -:**
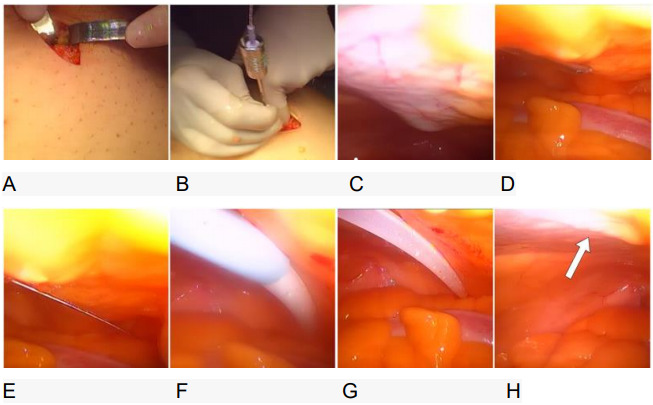
(A) Periumbilical incision; (B) insertion of the cannula of Veress needle with the guide wire of the kit; (C) the Veress needle progressing at least 6 cm caudally in the pre-peritoneal space; (D) needle enters the peritoneum; (E) guide wire enters the cavity; (F) the dilator with the seat is introduced through the tunnel; (G) catheter placed in the pouch of Douglas; and (H) pre-peritoneal tunnel is visible.

A 2 cm paraumbilical left incision below the umbilicus is made, followed by blunt dissection of the subcutaneous tissue until the anterior fascia of the rectus muscle is reached (Figure 1A). The stillet of the Veress needle is removed, and the outer cannula is inserted with the guide wire of Seldinger catheter kit (Figure 1B).

The Veress needle is introduced through the rectus muscle directed to the pelvis, making a tunnel of approximately 6 cm before entering the abdominal cavity (rectus tunnel sheath) - the same space is used for total extraperitoneal laparoscopic inguinal hernia repair (Figure 1C and 1D). Direct visualization of epigastric inferior vessels is important to avoid injuries.

The guide wire is advanced into the abdomen and the Veress needle is removed (Figure 1E). A dilator and the 16 Fr peel-away sheath are advanced over the guide wire into the tunnel and then to the abdominal cavity. The wire and dilator are removed and the catheter is advanced through the rectus tunnel sheath. The intraperitoneal segment is advanced until the deep cuff is immediately above the rectus sheath (in the subcutaneous tissue). The peel-away sheath and stylet are removed and the catheter position is checked (Figure 1F). The catheter tip should be placed in the pouch of Douglas (Figure 1G). The catheter entrance and tunnel are visible after the placement (Figure 1H). The catheter is rinsed and flushed with saline before the insertion. The inflow and outflow will be tested with at least 500 mL of saline solution with the patient in a neutral position. Approximately 350 mL of saline solution is left in the abdomen and catheter heparinized. After emptying the pneumoperitoneum, a subcutaneous tunnel is created with the tunneling stylet of the kit with the placement of the distal cuff subcutaneously, 2 cm from the exit site. The umbilicus port is removed and the supraumbilical fascia and skin are closed.

The kit used was a modified Seldinger peritoneal catheter kit with one 42 cm straight PD catheter and 2 dacron cuffs, syringe, scalpel 11, pull-apart sheath/dilator, J/straight stainless steel guidewire, and a tunneling stylet, gauze sponges, adapter, clamp, and cap.

After 14 days, the catheter testing was done by a dialysis nurse. Of the 16 patients who underwent laparoscopic insertion of a straight Tenckhoff catheter, 9 were men and 7 were women. The average age was 57 years (22-80). The mean operative time was 27 min (not included anesthesia induction). Among the 16 patients, 12 had a history of previous abdominal surgery and 1 required adhesiolysis due to a midline bowel adhesion and, therefore, an additional port was necessary.

## RESULTS

The mean follow-up period was 11 months (3-24). One patient died during the follow-up period due to other medical problems; one underwent renal transplantation some weeks after the implant; and one required an additional port for adhesiolysis due to previous abdominal surgeries. The other 13 had no problems related to catheter function. No postoperative abdominal wall hemorrhage, early leaks, hernias, or catheter migration occurred. One patient presented with a tunnel infection 11 months after implantation. No peritonitis was observed during the follow-up.

## DISCUSSION

PD and hemodialysis are dialysis options. PD was used for the first time in 1959. Henry Tenckhoff described in 1968 a catheter that was inserted using an open surgical technique. PD uses the peritoneum as an exchange membrane and offers the possibility of patients being treated at home^27^. Although not as widely used as hemodialysis, PD affords greater patient autonomy and quality of life than in-center hemodialysis. Some studies show that patient satisfaction has been significantly higher in PD patients^17^.

A successful PD with longer catheter survival highly depends on the method of insertion. PD catheter insertion can be accomplished by several different techniques. It is usually placed into the peritoneal cavity either by surgical technique (open surgery or laparoscopic-assisted) or by percutaneous technique (Seldinger or modified Seldinger technique), with or without fluoroscopic guidance^18^ ^,^ ^24^. The outcome of percutaneous implanted catheters, which were inserted by a trained nephrologist, was not demonstrated to be inferior as compared with the traditional surgical approach (open surgery)^4^.

The introduction of laparoscopy was a great advance in the implantation of PD catheters. It is associated with several advantages. The literature amply demonstrates the benefits of minimally invasive surgery^5^ ^,^ ^23^ ^,^ ^25^. Since the early 1990s, laparoscopy has been applied by many adult and pediatric surgeons for the insertion of PD catheters as well as for salvage of malfunctioning catheters^15^.

Surgical laparoscopy uses either a basic or advanced approach to provide PD access^11^. When the laparoscope is used only to witness the catheter tip position (simple or basic laparoscopy), the outcomes are no different from any other catheter insertion method^7^. Conversely, advanced laparoscopy was associated with a significant superior outcome in comparison with open insertion and basic laparoscopy^26^.

Recently, various advanced laparoscopic techniques for catheter placement have been investigated for better results and to minimize omental wrapping and catheter dislocation. Some of the advanced techniques described are rectus sheath tunneling, omentopexy, adhesiolysis, epiploectomy, salpingectomy apendicetomy, colopexy, pelvic fixation and diagnosis, and simultaneous repair of previously undiagnosed abdominal wall hernias ^1^ ^,^ ^6^ ^,^ ^7^ ^,^ ^9^ ^,^ ^14^ ^,^ ^16^ ^,^ ^19^ ^,^ ^22^.

RST, also described as extraperitoneal or preperitoneal tunneling, has been used by many authors as a way to maintain pelvic orientation and prevent catheter migration. RST consists of the creation of a long musculofascial tunnel in a craniocaudal direction, thus maintaining pelvic orientation of the catheter tip^6^ ^,^ ^10^ ^,^ ^19^ ^,^ ^22^. A detailed technique of rectal sheath tunneling has been described by Crabtree^8^ ^,^ ^10^. The catheter passes through a perpendicular passage for a short distance (4-6 cm) through the muscle. They described the use of a disposable bladeless trocar to make the tunnel^7^. Keshvari et al.^18^ described a new laparoscopic trocar for insertion of PD catheter and proper rectus sheath tunneling, because the previously used Tenckhoff trocar showed some disadvantages during a laparoscopic procedure, including the passage of insufflated gas through the trocar (lack of a proper valve mechanism) and difficulty in RTS due to the short length of the trocar.

Several studies show better outcomes with the RTS technique, with less complication of peritonitis, malposition, hernias, outflow obstruction, and leakage^3^. Attaluri et al.^1^ clearly showed a significant improvement in PD catheter function using omentopexy and rectus sheath tunneling. Keshvari et al.^19^ also found that a long preperitoneal tunnel fixes the extraperitoneal catheter without suturing, which can make this technique more effective in preventing catheter migration and reducing omental wrapping. Gultekin et al.^13^ described accurate placement, preperitoneal fixation, and immediate use of the catheter for routine PD and a decrease in outflow obstruction over long-term follow-up. RTS avoids the need for suturing the catheter tip to a pelvic structure. Suture fixation can be associated with difficulty in catheter removal as well as being a potential cause of internal hernia or adhesion^12^. Suture fixation may also impair the natural ability of the catheter to float to the largest area of PD fluid. Bar-Zohar et al.^2^ and Lu et al.^21^ showed a relatively high catheter dysfunction rate after suture fixation. In addition to requiring extra ports and time to perform, 6-9% of pelvic sutures erode from the tissues during the short-term resulting in an incidence of catheter tip migration^2^ ^,^ ^14^.

The results of a systematic review and meta-analysis reinforce the notion that advanced laparoscopic PD catheter insertion using RST in all cases, along with selective omentopexy and selective adhesiolysis, is associated with superior outcomes compared with basic laparoscopy and open insertion, in terms of both catheter dysfunction rate and overall catheter survival^26^.

The technique described in this study is a simple alternative for laparoscopic placement of PD catheter with RST using only the camera port, the Veress needle, and a modified Seldinger catheter kit, using a pull-apart sheath for the tunnel and introduction, and it is very reproducible.

There are some advantages of this technique. First, it does not need a second trocar, so it avoids excessive port sites. It can be done using only the normal laparoscopic material easily available, the Veress needle, and the catheter kit. It also reduces costs, because the kit already contains an 11 disposable scalpel, gaze sponges, a tunneling stylet, and the catheter itself. The Veress needle was chosen because it is longer than the 7 cm needle introducer of the catheter kit.

Another advantage is that the dilator and sheath peel apart are thinner than other trocars and devices described, which may reduce the chance of hernias or catheter leak. It also maintains pneumatic competence and visibility during laparoscopy.

In this technique, the deep cuff of the catheter was placed just above the anterior rectus sheath (in the subcutaneous tissue). Although, traditionally, it is preferably placed within the rectus muscle, in a series with a large number of patients, there were no adverse effects directly connected to positioning the deep cuff subcutaneously^16^ ^,^ ^21^. It is less traumatic and makes the removal of the catheter easier. In our experience, the removal is usually done under local anesthesia as an ambulatorial procedure.

Finally, other proceedings and techniques like omentopexy, adhesiolysis, and pelvic fixation can also be performed simultaneously by adding other ports, if necessary, after abdominal cavity exploration.

## CONCLUSIONS

The technique described is simple, reproducible, and safe. It can be done by a general surgeon and has good results in catheter function, with a low complication rate and high catheter survival rate. The technique does not require any special device or trocar and avoids excessive port sites.
